# Corona viruses: reaching far beyond the common cold

**DOI:** 10.4314/ahs.v21i1.27

**Published:** 2021-03

**Authors:** Kathleen M Coerdt, Amor Khachemoune

**Affiliations:** 1 Georgetown University School of Medicine, Washington, DC; 2 SUNY Downstate, Department of Dermatology, Brooklyn, NY

**Keywords:** Coronavirus, common cold, severe respiratory disease, COVID-19

## Abstract

**Background:**

Human coronaviruses (HCoVs) are one of the most common causes of the “common cold”. Some HCoV strains, however, can cause fatal respiratory disease. Some examples of these diseases are severe acute respiratory syndrome (SARS), Middle East respiratory syndrome (MERS), and Coronavirus Disease 19 (COVID-19). This article will review the etiology, clinical features, diagnosis, and management of HCoVs.

**Methods:**

A systematic literature review was performed using the terms “human coronaviruses”, “MERS-CoV”, “SARSCoV”, “SARS-CoV2”, “COVID-19”, and “common cold” in OVID MEDLINE, PubMed, and Cochrane Library.

**Findings:**

Most HCoVs cause mild upper respiratory infections which resolve with supportive care and no sequelae. In recent decades, however, there have been outbreaks of novel HCoVs that cause more severe disease. This is largely due to HCoVs having large genomes which undergo frequent recombination events, leading to the emergence of novel and more virulent strains of the virus. These severe respiratory illnesses can lead to acute respiratory distress requiring invasive intervention, such as mechanical ventilation. These severe infections can lead to long-lasting sequelae in patients. Scientists continue to investigate potential treatments for these viruses, though supportive care remains the gold standard. Scientists have succeeded in developing numerous vaccines for the SARS-CoV-2 virus, and ongoing data collection and analysis will shed even more light on the next steps in fighting the COVID-19 pandemic.

**Conclusion:**

Due to the frequency of recombination events and the subsequent emergence of novel strains, HCoVs are becoming more prevalent, making them a global health concern as they can lead to epidemics and pandemics. Understanding the epidemiology, etiology, clinical features, diagnosis, and management of HCoVs is important, especially during this worldwide pandemic.

## Introduction

Corona viruses (CoVs) are one of the most common causes of the “common cold”.^1^,^2^,^3^ CoVs can also cause pneumonia and severe respiratory disease.^3^,^4^,^5^ CoVs are enveloped, positive-sense, single-stranded RNA viruses.^2^,^4^,^5^,^6^ They have the largest genome of the known RNA viruses, being between 26–32 kilobases in size.^2^,^7^ CoVs have spike projections which, under the microscope, resemble a crown; corona means “crown” in Latin, and it is how the virus got its name.^2^ The spike projections allow for attachment to cellular receptors, which allows the CoVs to be internalized into host cells by direct fusion with the plasma membrane or by endocytosis. 1,6,7,8

CoVs can infect animals and humans; animals often serve as the reservoir and intermediate host of the virus.^1^,^4^,^6^,^7^ CoVs are constantly evolving with new strains of the virus emerging.^2^,^4^ Seven CoVs have been found that infect humans; the human CoVs (HCoVs) are HCoV-229E, HCoV-NL63, HCoV-OC43, HCoVHKU1, severe acute respiratory syndrome CoV (SARSCoV), Middle East respiratory syndrome-CoV (MERSCoV), and SARS-CoV-2 (Table 1).^1^,^2^,^4^,^5^,^6^

**Table 1:** **Comparison of the seven human coronaviruses.** The origin group, distribution, seasonality, and incubation period of each are shown

Strain	Group	Distribution	Season	Incubation
HCoV-229E	Alpha	Globally	Winter	2–5 d
HCoV-NL63	Alpha	Globally	Summer/Fall	2–5 d
HCoV-OC43	Beta	Globally	Winter	2–5 d
HCoV-HKU1	Beta	Globally	Spring/Summer	2–5 d
SARS-CoV	Beta	Globally	Winter	2–14 d
MERS-CoV	Beta	Arabian Peninsula	Variable	2–15 d
SARS-CoV-2	Beta	Globally	Winter?	2–14 d

In the last two decades, there have been outbreaks of novel HCoVs, which have caused severe respiratory disease.^4^ The severe acute respiratory syndrome (SARS) and Middle East respiratory syndrome (MERS) outbreaks were causd by SARS-CoV and MERS-CoV, respectively. Most recently, a novel CoV, SARS-CoV-2, emerged from China.^2^,^9^ It causes Coronavirus Disease 19 (COVID-19), and it quickly spread throughout the world.^9^ All three of these outbreaks have caused severe respiratory disease and death in many patients.^4^

Therefore, HCoVs should not be underestimated. Though they most commonly cause a self-limited, upper respiratory tract infection, they can be deadly.^1^,^2^,^4^ In this review, we will explore the epidemiology, etiology, clinical manifestations, and management of HCoV infections.

## Epidemiology

HCoVs are estimated to cause between 15–30% of common colds in adults.^1^,^2^ HCoVs are usually spread via respiratory droplets and contact routes.^10^ They are typically associated with upper respiratory tract infections but can also cause lower respiratory tract infections and pneumonia.^1^,^3^ HCoVs infect all ages and generally peak in the winter months in temperate climates.^1^,^2^ It is estimated that HCoVs cause epidemics every 2 to 3 years.^1^

The most common HCoV strain is HCoV-229E, which was discovered in 1966. It is found globally, peaks in winter months, and can infect all ages.^1^,^2^ HCoV-OC43 also peaks in the winter and is found globally.^1^ HCoVNL63 was first discovered in 2004 and HCoV-HKU1 in 2005; both are found across the world.^1^,^5^ HCoV-NL63 is responsible for about 5% of common colds. It primarily infects children less than five years old but can infect all ages.^1^ Most infections occur in the summer and fall rather than the winter.^1^ Likewise, HCoV-HKU1 typically occurs in children under five years, though it can also infect adults.^3^ HCoV-HKU1's seasonality is less studied but has been found to be most prevalent in hospitalized patients in the spring and summer months.^5^

SARS-CoV emerged from China in 2002, and MERS-CoV originated in Saudi Arabia in 2012, causing the SARS and MERS outbreaks, respectively.^2^ SARS arose in the winter, spread over 37 countries, and took 775 lives, leading to a case fatality rate (CFR) of 9% (Table 2).^2^,^11^ MERS was most prevalent in the Arabian Peninsula without clear seasonality.^2^,^6^,^12^,^13^ It has an estimated CFR of 36%.^2^,^6^,^12^ In December 2019, several cases of unexplained pneumonia were reported in Wuhan, China.^14^ A novel HCoV, SAR-CoV-2, was isolated in these patients.^14^ The disease caused by SAR-CoV-2 was deemed COVID-19.^9^,^14^ COVID-19 spread rapidly across the world and was declared a pandemic by the World Health Organization on March 11, 2020.^14^ On March 11, there were 126,000 cases of COVID-19; by April 4, there were over 1,196,000 cases.^15^ Initial data shows that SARS-CoV-2 is more contagious than SARSCoV. 7 Thus, heighted infection prevention and control were put in place over the globe to slow the spread.^14^ Additionally, there have been reports of viable SARSCoV-2 in stools of COVID-19 patients and viral RNA in sewage waters.^10^ Patients may continue to shed the virus in feces even after respiratory symptoms disappear. 10 Thus, some researchers speculate that fecal-oral transmission is possible.^10^ Further research is needed on the evidence and relevance of possible fecal-oral transmission. 10 If this mode of transmission is possible, prevention of disease will require adequate sanitation and clean water initiatives around the globe, particularly in developing countries.^10^ Interestingly, COVID-19 is not particularly prevalent in Africa; since it is a developing country, some may speculate that disease rates would be higher in Africa if fecal-oral transmission is possible. Disease prevalence may remain low, however, due to less international travel and more secluded communities in Africa. A recent Chinese study on COVID-19 by Wu et al. reported an overall CFR of about 2.3% for COVID-19.^4^ The most recent worldwide data has a CFR around 5.39%.^15^ This, however, only takes into account confirmed cases of COVID-19. Over 80% of the cases reported had mild symptoms.^4^ Thus, there is likely a large asymptomatic infected population, causing the CFR to be overestimated.^16^ Unfortunately, the CFR increases to 8% in those between 70–79 years and to 14.8% in those 80 years and older, putting the elderly population at a much higher risk for severe disease.^4^,^15^

**Table 2:** **Comparison of the three human coronaviruses which cause severe respiratory disease in patients.** The epidemiology and clinical manifestations of severe acute respiratory syndrome (SARS), Middle East respiratory syndrome (MERS), and Coronavirus Disease 2019 (COVID-19) are compared

	SARS	MERS	COVID-19
**Epidemiology**			
Virus	SARS-CoV	MERS-CoV	SARS-CoV-2
Origin	China	Saudi Arabia	China
Distribution	Global	Arabian Peninsula	Global
Reservoir	Bats	Camels	Bats
Intermediate Host	Masked palm civets	n/a	Minks?
Incubation Period	2–14 days (up to 21 days)	2–15 days	2–14 days (up to 27 days)
**Clinical**			
Primary symptoms	Pneumonia, respiratory distress	Pneumonia, respiratory distress	Cough, fever, shortness of breath, myalgias, pneumonia, respiratory distress
Extrapulmonary symptoms	Diarrhea	Acute renal failure, sepsis, diarrhea	Acute cardiac injury
Imaging	CXR: ground-glass opacities, ARDS	CXR: focal to diffuse infiltrates, consolidation, ARDS	CT: ground-glass shadow
Diagnosis	RT-PCR	RT-PCR	RT-PCR
Treatment	Supportive	Supportive	?
CFR	10%	36%	2–5%?

## Etiology

There are four types of CoVs: alpha, beta, gamma, and delta. Only alpha-CoVs and beta-CoVs have been shown to infect humans, and most are of bat origin.^2^,^4^ HCoVs 229E, OC43, NL63 and HKU1 are adapted to humans and do not appear to have animal reservoirs. 2 SARS-CoV and MERS-CoV, however, are not well adapted to humans; they are spread by animal reservoirs and intermediate hosts (Table 2).^2^,^4^ SARSCoV stemmed from a wild horseshoe bat reservoir in China in 2003.^17^ Masked palm civets from live animal markets in Guangdong, China were the intermediate hosts, spreading the disease to humans.^4^,^17^ MERS-CoV originated in camel reservoirs in Saudi Arabia in 2012, and the disease may have spread directly from camels to humans.^18^ There is some evidence of the MERS-CoV genome in bats, though the genome likely underwent recombination in camels before infecting humans.^18^ It is unknown whether an intermediate host exists for MERS.^4^,^18^ SARS-CoV-2 originated in a wet market in Wuhan, China.^4^ It shares 70% similarity to the SARSCoV species.^4^ Like many other HCoVs, it likely originated in bats as it shares 96% homology with bats, though this requires further confirmation.^4^ It has also been speculated that minks and pangolins are the intermediate hosts of this novel virus.^4^ The increasing amount of human-animal contact increases the risk of outbreaks, such as the mot recent outbreak of SARS-CoV-2.^4^

Furthermore, CoVs undergo extensive genetic recombination and evolution, and resultant recombinant strains may be more virulent and deadly to humans.^2^,^4^,^6^ The estimated mutation rates are moderate-to-high compared to other single-stranded RNAs.^2^ The large CoV genome allows for great diversity in intraspecies and interspecies variability and in the emergence of novel CoVs.^2^ Recombination occurs primarily at the replication phase of the virus's life cycle.^2^ Though the exact recombination mechanism remains unclear, the crossing over points appear to be random, leading to a plethora of possibilities for genomes.^2^

For example, SARS-CoV emerged via horizontal transmission and genetic recombination between an alpha-CoV and a gamma-CoV, and MERS-CoV underwent multiple recombination events, which allowed both of these strains to infect humans.^2^ The high frequency of recombination allows for generation of novel and more virulent HCoVs.^2^ Such mutations can lead to epidemics and pandemics across the globe, as we are seeing now with SARS-CoV-2.^2^

## Clinical presentation

HCoVs typically cause mild upper respiratory tract infections. 1,19 They can also cause lower respiratory tract infections and gastrointestinal symptoms in some patients. 2 Fever, rhinorrhea and cough are manifestations of upper respiratory tract infections, whereas productive cough and dyspnea are common lower respiratory tract infection symptoms.^2^ The severity of disease ranges from mild and self-limited to severe and life-threatening. 2,19 Mild, self-limited infections typically manifest as the common cold.^2^ HCoVs are responsible for 15–30% of common colds.^1^,^2^ More severe cases can manifest as bronchitis, pneumonia, and acute respiratory distress syndrome (ARDS).^2^ Some infections also have accompanying renal failure or neurologic involvement.^1^,^2^

HCoV-229E^19^ generally causes a self-limited common cold; symptoms include malaise, headache, sneezing, rhinorrhea, and sore throat.^2^,^19^ A minority of patients develop fever and cough.^2^ The average incubation time is between 2–5 days, and the illness lasts 2–18 days.^2^ HCoV-229E symptoms are virtually indistinguishable from those caused by rhinovirus and influenza A.^2^ HCoV-OC43 presents similarly to HCoV-229E.^2^ Both generally cause infection in immunocompetent adults.^2^ HCoV-NL63 and HCoV-HKU1, on the other hand, primarily infect young children, elderly, and immunocompromised patients.^2^,^19^ HCoV-NL63 symptoms typically include fever, cough, rhinorrhea, tachypnea, and hypoxia.^2^ Patients with HCoV-NL63 may develop croup.^2^ HCoV-HKU1 presents similarly to the other HCoVs with fever, rhinorrhea, and cough.^2^ It is typically a mild, self-limited infection but can cause severe respiratory disease and pneumonia in young children.^19^ It is also associated with a high risk of seizures.^2^

The remaining three HCoVs can present more dramatically with severe respiratory disease.^17^,^19^ SARS initially presents with fever, chills, myalgia, malaise, and headaches. 2 A nonproductive cough with dyspnea, hypoxia, and respiratory distress follows 5–7 days after the onset of symptoms.^1^,^2^ Severe respiratory distress may result in death. Symptoms may also involve the gastrointestinal tract, liver, kidney, and brain.^2^ Diarrhea is seen in 30–40% of cases.

MERS symptoms range from asymptomatic to severe pneumonia with acute respiratory distress, septic shock, and renal failure.^2^ The course typically begins with flu-like symptoms: fever, chills, myalgia, cough, sore throat and arthralgias.^1^,^2^,^12^ It quickly worsens to dyspnea and pneumonia.^2^,^12^ About one-third of patients have gastrointestinal manifestations, such as diarrhea and vomiting.^2^ Renal failure is seen in a subset of patients with the disease, which is unique to this HCoV.^2^,^5^ Some patients may also develop myocarditis.^11^ The majority (75%) of patients who contract MERS have at least one comorbidity, such as diabetes, cardiovascular disease, obesity, renal failure, and/or immunodeficiency.^2^,^12^,^17^

COVID-19 typically presents as an acute respiratory infection.^4^,^9^.^20^ It can present in numerous ways – from mild, asymptomatic disease to rapidly progressive, fatal disease.^20^ The majority of cases are mild in immunocompetent patients, causing fever, cough, myalgias and shortness of breath.^4^ Pneumonia occurs in some patients.^4^ Lymphopenia and thrombocytopenia may also occur.^4^,^21^ Severe disease, however, can lead to respiratory distress requiring intubation, acute cardiac injury, secondary infection, and even death.^4^ Patients who are older in age, have an underlying respiratory disease, have multiple comorbidities (ie obesity, diabetes, cardiovascular disease), and/or are immunocompromised are more likely to have severe disease.^4^,^20^ The mean incubation period has been found to be about 5.2 days.^4^ The incubation period until dyspnea is 5 days, until hospital admission is 7 days, and until acute severe respiratory syndrome is 8 days.^4^ The CFR is lower for COVID-19 than for SARS and MERS.

A plethora of skin findings have been associated with COVID-19.^22^,^23^,^24^ Thrombocytopenia can lead to petechiae.^21^ Acral pseudo-chilblain lesions, vesicular eruptions, widespread and localized urticarial lesions, erythematous maculopapular eruptions, and livedoid/necrotic lesions also have been reported in studies of patients with COVID-19.^22^,^23^ Most popularly known are the acral lesions, also called “COVID toes”.^23^,^24^ These toes appear red and violaceous with accompanying itching, swelling, and/or pain.^24^ The pain can be debilitating, causing a limp or an inability to walk in some patients.^24^ These toes manifestations are hypothesized to be due to thrombotic complications of the disease, but further research is necessary on this.^24^

## Diagnosis

All HCoVs have been detected by RT-PCR, and this is the gold-standard for diagnosis.^1^ They can also be detected with intensive cultures, but PCR has a greater sensitivity than cultures.^1^

Imaging can help diagnose the severe respiratory infections. On chest X-ray (CXR), SARS appears as groundglass opacities or ARDS.^1^ ARDS generally appears as bilateral homogenous opacities on CXR; computed tomography (CT) tends to show bilateral opacities in dependent lung regions in ARDS.^25^ MERS typically starts with unilateral focal lesions and progresses to multifocal or bilateral involvement on CXR.^1^ Chest CT shows bilateral basilar involvement in MERS.^1^ On CXR, COVID-19 may shows multilocular, patchy infiltrates in the lung periphery.^4^ On chest CT, COVID-19 presents with bilateral ground-glass opacities.^4^

## Management

Most HCoVs are self-limited infections. Oral or intravenous hydration, rest, and supportive care are key during recovery.^26^ For the severe HCoV infections, however, more intensive treatment may be necessary.^26^ For example, respiratory support, functional organ support, plasma exchange, and extracorporeal membrane oxygenation (ECMO) may be used.^4^ For SARS, ribavirin, interferons, and/or corticosteroids were speculated to be beneficial in patients; for MERS, ribavirin and/or interferon were proposed.^1^,^17^ Though antivirals and immunomodulators have been used for SARS and MERS, there is no evidence in randomized controlled trials showing these to be effective.^1^,^17^ Evidence-based treatments and vaccines continue to be investigated for both SARS and MERS.^17^ Thus, supportive measures remain the mainstay of therapy.^1^,^17^ Oxygen supplementation and mechanical ventilation may be required.^1^,^17^,^23^ Dialysis may become necessary if patients progress to acute renal failure.^1^,^4^

For the novel COVID-19, there is no specific antiviral treatment.^7^ Researchers are currently studying various drugs and biologics in hopes of finding a treatment to save lives.^7^ A study in France by Gautret et al. evaluated the effect of hydroxychloroquine on SARS-CoV-2 viral loads.^16^ The study used 600 mg hydroxychloroquine (200 mg three times per day) in confirmed COVID-19 patients, and azithromycin was added for some patients based on presentation.^16^ Twenty cases were treated and showed a significant reduction of the viral load by six days post-diagnosis in comparison to controls.^16^ This suggests a shortened average carrying duration, which may reduce transmission.^16^ Azithromycin significantly increased efficiency of viral elimination, showing a synergistic relationship between the two medications.^16^ While the initial data looks promising, the limitations of this study were a small sample size and limited follow-up.^16^ Thus, more data is necessary to determine the efficacy of hydroxychloroquine on COVID-19.

Researchers also have been investigating remdesivir as potential treatment for COVID-19.^27^ Remdesivir was initially developed for Ebola-infected patients.^27^ It is a nucleotide prodrug, which inhibits viral RNA replication. 27 Remdesivir was shown to inhibit SARS-CoV and MERS-CoV replication in cell cultures with human airway epithelial cells, and a more recent study showed that remdesivir is active against SARS-CoV-2 in cell culture. 27 A preliminary report of an ongoing randomized controlled trial in hospitalized adult patients with COVID-19 showed a shortened time to recovery in patients treated with remdesivir versus placebo.^28^ For this reason, the United States Food and Drug Administration (FDA) has made remdesivir available under an emergency-use authorization for children and adult patients with severe COVID-19.^28^ Though the drug appears beneficial to patients with severe disease who require oxygen therapy, remdesivir is not sufficient to fight this virus on its own.^28^ The mortality rate remains high despite remdesivir therapy, so researchers continue to race the virus in hopes of finding a treatment and creating a vaccine for future prevention.^28^

With respect to controlling the spread of infection, isolation is necessary for patients with suspected or confirmed SARS, MERS or COVID-19.^26^,^29^ Handwashing is vital in preventing the spread of the disease along with limiting person-to-person contact.^29^ Proper precautions should be taken for suspected and confirmed cases in the hospital.^26^ These include standard, contact, and droplet precautions. Aerosolized precautions should be employed when doing invasive aerosol-producing procedures, such as intubating.^1^ For COVID-19, healthcare workers are recommended to use an N95 respirator mask, eye protection (goggles or face shield), gloves, and gowns.^29^ Unfortunately, N95 masks were in short supply at the beginning of the pandemic. If healthcare providers do not have access to an N95, a surgical mask is recommended even though it offers less protection.^29^

Finally, for a patient to be considered “cured” of COVID-19, a nasopharyngeal swab should be performed every 48–72 hours.^26^ When the SARS-CoV-2 RT-PCR is persistently negative, a patient is no longer infected.^26^

## Conclusion

HCoVs are widespread across the globe. Due to their extensive genetic recombination along with the increasing amount of human to animal contact, novel virulent strains of HCoVs can emerge, leading to epidemics and pandemics. Most infections are mild, self-limited upper respiratory tract infections, though severe, life-threatening respiratory distress can occur. Both mild and severe infections are best managed with supportive care, though research is ongoing in hopes of finding a treatment. Through the hard work of scientists during the COVID-19 pandemic, numerous vaccines against SARS-CoV-2 have been produced. As regulatory bodies continue to approve these vaccines and distribution continues, there is hope for protection against the virus.

## Appraisal and Word of Wisdom

The world has seen epidemics and pandemics more devastating than the ones caused by coronaviruses. One of these is the plague of 1340s where about half of Europe's inhabitants perished due to the effect of Yersinia pestis. One unique aspect of COVID-19 is its novelty and our limited understanding of both its virologic and clinical features. Additionally, the hype that media has put to it has created a worldwide state of panic and crisis at all levels affecting the majority of countries and all sectors of their populations. As we make progress in understanding the new virus, the spectrum of disease it causes, and the management options, we predict that we will be talking about it in the past as we are now talking about SARS, the plague, and cholera epidemics and pandemics.
